# Some Anti-Plague Measures

**Published:** 1919-12-13

**Authors:** 


### Some Anti-Plague Measures.

The whole question of means by which plague extension can be prevented is one on which many pages may be, and have been, written, and it is the object of this note merely to draw attention to certain simple ways in which portions at least of the desired end may be attained. One point to be remembered is that bacilli can remain in the bodies of recovered patients for a considerable time—three weeks at least—so that, in spite of the usual quarantine period of ten days, to be on the safe side convalescents should be isolated for a whole month. Also care in destruction and disinfection of possibly infected fomites is more important than some people imagine, in that the bacilli have under favourable circumstances great vitality outside the human body. In connection with rat destruction it is perhaps worth noting that domestic cats and dogs must be kept rigidly away from infected cases for obvious reasons. And again, there are a number of simple facts with regard to trapping, etc., which are not generally known. Mention may be made of the value of tomatoes, with or without the addition of phosphorus paste, as a bait for rats. According to official directions value is attached to the use of baited birdlime spread on boards, while extract of squills, of which one-tenth of a milligram will kill a rat, and barium carbonate have also been successfully employed. To prevent pneumonic plague transference the freest ventilation is necessary, for it is found that in close, moisture-saturated atmospheres the infections take place most readily.

